# Evaluating Racial Disparities in Access to Common Pediatric/Congenital Transcatheter Interventions

**DOI:** 10.1016/j.jacadv.2025.102158

**Published:** 2025-09-23

**Authors:** Michael L. O’Byrne, Kevin F. Kennedy, Ryan Callahan, Yoav Dori, Jonathan J. Rome, Christopher L. Smith, Jie Tang, Matthew J. Gillespie, Christopher M. Janson

**Affiliations:** a Division of Cardiology The Children's Hospital of Philadelphia and Department of Pediatrics Perelman School of Medicine at The University of Pennsylvania, Philadelphia, Pennsylvania, USA; b Leonard Davis Institute The University of Pennsylvania, Philadelphia, Pennsylvania, USA; c Cardiovascular Outcomes, Quality, and Evaluative Research Center, Perelman School of Medicine at the University of Pennsylvania, Philadelphia, Pennsylvania, USA; d Mid America Heart Institute St. Luke’s Health System, Kansas City, Missouri, USA

**Keywords:** atrial septal defect, congenital heart disease, health services research, outcomes, patent ductus arteriosus

## Abstract

**Background:**

Disparities in outcomes for congenital heart surgery patients have been observed. To our knowledge, these disparities have not been studied in pediatric/congenital interventional cardiology.

**Objectives:**

The purpose of this study was to determine whether Black race or Hispanic ethnicity is associated with disparities in diagnosis, referral, and/or access to catheterization.

**Methods:**

A multicenter cohort study of patients ≤18 years who underwent: 1) device closure of atrial septal defect (ASD); 2) device closure of patent ductus arteriosus; 3) balloon pulmonary valvuloplasty (BPV); and 4) balloon aortic valvuloplasty (BAV) at centers contributing to the IMPACT registry 6/1/2016-12/31/2022. The associations between race-ethnicity and age at procedure and other procedure-specific markers of disease severity were evaluated. Also, the associations between race-ethnicity and the risk of adverse events and the proportion of patients treated at high-, medium-, and low-volume centers for each procedure were evaluated.

**Results:**

In total, 20,632 subjects were studied (34% ASD, 48% patent ductus arteriosus, 14% BPV, and 5% BAV). Race-ethnicity was associated with older age for ASD and BPV (*P* < 0.05) and more severe disease for BPV and BAV (*P* < 0.05). The proportion of Black and Hispanic patients was higher in low-volume than in high-volume centers (*P* < 0.05 for all procedures).

**Conclusions:**

Minority race-ethnicity was associated with older age at referral and more severe disease, implying disparities in referral and/or access and higher likelihood of treatment at low-volume centers. Efforts to understand and mitigate disparities in this population should focus on screening, diagnosis, and access.

Disparities in outcomes in patients belonging to racial and ethnic minority groups have been documented in patients receiving medical and surgical care.^1^, ^2^, ^3^, ^4^, ^5^, ^6^, ^7^, ^8^, ^9^, ^10^, ^11^ These disparities have been observed in congenital cardiology, where Black and Hispanic children consistently experience worse outcomes after congenital heart disease surgery compared to non-Hispanic White children.^3^^,^^4^^,^^12^, ^13^, ^14^, ^15^, ^16^ To our knowledge, no studies to date have evaluated whether analogous disparities are seen after pediatric/congenital catheterization laboratory (PCCL) procedures.

The potential mechanisms underlying racial/ethnic disparities identified in previous research^5^^,^^8^^,^^17^^,^^18^ include: 1) disparities in referral; 2) disparities in timing of referral; and 3) disparities in access to higher volume programs (Central Illustration A). In PCCL patients, failure or delays in diagnosis could potentially lead to delays in referral manifest as older subjects and/or higher proportions of patients with severe disease. Disparities in access to regional or national centers of excellence might also incur greater procedural risk. The presence of any of these disparities could increase the risk of harm in disadvantaged populations. Mitigating these disparities and providing optimal outcomes to all patients would be improved by identifying the underlying mechanism.

**Central Illustration:**
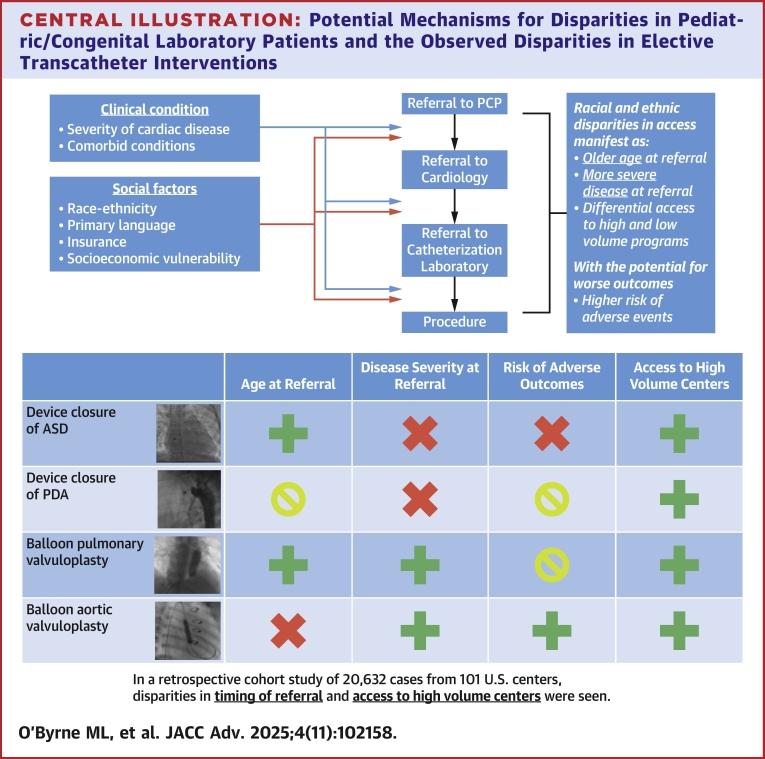
Potential Mechanisms for Disparities in Pediatric/Congenital Laboratory Patients and the Observed Disparities in Elective Transcatheter Interventions (Top) Reaching the catheterization for treatment of nonemergent defects requires navigating multiple levels of the health care system. Each of these steps is potentially affected by both the patient’s severity of disease and social factors, specifically race-ethnicity, primary language, insurance, and socioeconomic resources/vulnerability. We hypothesize that if disparities are present disadvantaged groups will be older at referral for their procedures, present with more severe disease, and will be disproportionally treated at smaller volume centers. There may also be disparities in outcome (eg, adverse events). (Bottom) After adjusting for measurable confounders disparities (green crosses) are seen in age at referral, disease severity at referral, and risk of adverse outcomes. In some cases, point estimates suggested presence of disparities but the associations were not statistically significant (yellow zero). Areas where there was no evidence of disparity are also identified (red x). ASD = atrial septal defect; PCP = primary care physician; PDA = patent ductus arteriosus.

One obstacle to studying these disparities is the diversity of PCCL procedures. In some cases, timing of referral for PCCL procedures is dictated by clinical status (eg, interventions and palliations in critically ill neonates) or are tightly linked to surgical care and its quality (eg, postoperative interventions and their outcome). These types of cases are less likely to be informative about disparities in diagnosis, referral, and access. Also, because high-severity adverse event (AE) rates are low, there will be limited statistical power to evaluate disparities.^19^, ^20^, ^21^ Because of these factors, investigating disparities in PCCL referral requires a large cohort of homogenous procedures, the timing of which could plausibly be affected by differences in referral patterns from a broad representative sample of centers (to ensure applicability of their findings) with relevant patient and procedural data to address potential confounders. We used data from the IMproving Pediatric and Adult Congenital Treatment (IMPACT) Registry to overcome these obstacles by performing a multicenter retrospective cohort study. We hypothesized that racial/ethnic minority patients would be associated with worse access to PCCL services and that this would be observed as: 1) older age at presentation; and 2) higher proportions of patients with markers of severe disease in Black and Hispanic patients. A secondary goal was to evaluate if there were measurable differences in the likelihood of AEs associated with race and ethnicity. Identification of disparities in access is important to direct mitigation efforts and improve care of this vulnerable population.

## Methods

### Data source

The IMPACT registry is a clinical registry funded by the American College of Cardiology and managed by the National Cardiovascular Data Registry with data from 101 North American pediatric and general hospitals performing cardiac catheterizations in children and adults with congenital heart disease at the time of this analysis. Participating centers collect demographics, medical/surgical history, procedural information, and AE through hospital discharge on all patients undergoing cardiac catheterization. Data are recorded using standardized data elements and definitions. The database is subject to quality assurance standards.^22^ The current study used data from IMPACT v1 and v2. Analysis of deidentified data does not qualify as human subjects research as per the Common Rule (45 CFR 46.102(f)), and waiver of written informed consent and authorization for this study was granted by Advarra, the central Institutional Review Board for IMPACT. Because of existing data-use agreements, sharing of subject-level data is prohibited.

### Study design/population

A multicenter retrospective cohort study of cases performed at IMPACT centers between 6/1/2016 and 12/31/2022 was performed. Inclusion criteria for IMPACT centers were for >80% of cases to have race or ethnicity data recorded. For individual subject cases to be included, race or ethnicity data had to be recorded. The decision to include cases with either race or ethnicity because there was an expectation that a significant portion of Hispanic cases would not have race reported. Cases were taken from one of 4 case types: 1) device occlusion of atrial septal defect (ASD); 2) device occlusion of patent ductus arteriosus (PDA); 3) balloon pulmonary valvuloplasty (BPV); and 4) balloon aortic valvuloplasty (BAV). These 4 procedures are core procedures in IMPACT for which additional data about both outcomes and confounding are recorded. As a result, these procedures have been studied frequently and have well-defined conceptual models of covariates and their effects on outcomes.^23^, ^24^, ^25^, ^26^ For each case type, additional procedure-specific exclusion criteria were applied to ensure that within each group cases were comparable (Table 1).

**Table 1:** Inclusion and Exclusion Criteria

Centers	Inclusion Contributing Data to IMPACT Between 1/1/2011 and 12/31/2022 Race/Ethnicity Reported in >80% of Cases
Individual subject/cases	Inclusion Race/ethnicity reported Exclusion ECMO, LVAD, IABP in place at the start of procedure Outcome • Age at procedure • Death prior to discharge • Catastrophic adverse outcome • Major adverse outcome
Device closure of atrial septal defect	Inclusion • Age >365 d ≤ 18 y • Isolated single atrial septal defect • No other procedures performed and no prior catheterization or surgery • Indication: right ventricular volume overload Additional covariates • Ratio of systemic to pulmonary blood flow • Multifenestrated atrial septal defect • Tissue rim measurements performed • One or more deficient tissue rims Additional outcomes • Ratio of systemic to pulmonary blood flow • More than trivial residual shunt • Device embolization
Device closure of patent ductus arteriosus	Inclusion • Age ≤18 y • Isolated single patent ductus arteriosus • No other procedures performed and no prior catheterization or surgery • Indication: left ventricular volume overload or spontaneous bacterial endocarditis prophylaxis Exclusion • Age <365 d and prematurity Additional covariates • Ratio of systemic to pulmonary blood flow • Anatomic classification of ductus arteriosus • Procedural indication (volume overload vs endocarditis prophylaxis) Additional outcomes: • Ratio of systemic to pulmonary blood flow • More than trivial residual shunt • Device embolization • Device obstruction of a pulmonary artery • Device obstruction of the aorta
Balloon aortic valvuloplasty	Inclusion • Age ≤18 y • Age >30 d • Indication: high resting gradient or ventricular dysfunction Exclusion • Previous congenital heart disease surgery • Previous catheterization Additional covariates Procedural indication (high resting gradient vs ventricular dysfunction) Preprocedure gradient Aortic valve morphology (biscuspid vs other) Additional outcomes: Balloon rupture Postprocedure gradient Postprocedure gradient >50 mm Hg Moderate or greater postprocedure insufficiency
Balloon pulmonary valvuloplasty	Inclusion Age ≤18 Age >30 d Indication: high resting gradient or ventricular dysfunction Exclusion Previous congenital heart disease surgery Previous catheterization Additional covariates Procedural indication (high resting gradient vs ventricular dysfunction) Dysplastic pulmonary valve Preprocedure gradient Additional outcomes Balloon rupture Postprocedure gradient Postprocedure gradient >50 mm Hg

### Study measures

Race and ethnicity are separate domains and recorded independently in IMPACT. Patients can be identified as White, Black, American Indian/Alaskan Native, Asian, or Native Hawaiian/Pacific Islander. The registry allows a patient to be identified by more than race. The registry further requests that Asian and Native Hawaiian/Pacific Islander are further identified (eg, Native Hawaiian, Guamanian or Chamorro, Samoan, or other). Hispanic ethnicity is recorded separately along with a request to further identify patients of Mexican, Puerto Rican, Cuba, or other Hispanic/Latin/Spanish origin. We anticipated that many people identified in IMPACT as Hispanic would not have race listed consistently. This was confirmed in preliminary evaluation (data not shown). To facilitate analysis, race and ethnicity were combined into the following mutually exclusive categories non-Hispanic White, Hispanic (all races), non-Hispanic Black, non-Hispanic Asian, and other non-Hispanic (comprised of subjects identified as Native American, Native Alaskan, Native Hawaiian, Pacific Islander, and more than one race). The miscellaneous other-race group was used because the total populations of individual component groups were small. To our knowledge, racial and ethnic identity in IMPACT is based on the electronic medical record and not self-report, and as a result is subject to potential inaccuracies.^27^^,^^28^

For each subject, the following data were collected: age, sex, weight, height, body mass index (BMI) insurance payer (commercial, public, or other), and hemodynamic vulnerability (as defined previously).^21^^,^^29^, ^30^, ^31^ For specific case types, additional data were collected to evaluate the severity of illness for illness at presentation (Table 1, eg, for ASD cases the ratio of pulmonary to systemic blood flow (Qp:Qs) and presence of deficient tissue rims) and where available processes performed during the case (eg, for ASD cases was balloon sizing performed and were rim measurements performed). For each subject, distance traveled to reach the treating hospital was calculated as the distance between the geographic center of their home zip code to the location of the treating hospital. Distance was evaluated as a continuous variable. We also recorded the annual catheterization laboratory volume for each center averaged over the study period as a measure of program size.^21^^,^^32^, ^33^, ^34^ We did not seek to link zip code data to other social determinants of health (eg, median income or indices such as Childhood Opportunity Index).

For each case type, the following adverse outcomes were collected: death prior to discharge, catastrophic adverse outcome (death prior to discharge, initiation of mechanical extracorporeal membrane oxygenation, ventricular assist device, or intra-aortic balloon pump, repeat catheterization due to catheterization complication, vascular complication requiring treatment, and/or unplanned surgery due to catheterization complication), and composite major AE (any catastrophic AE, new arrhythmia, new heart valve regurgitation, cardiac tamponade, air embolus, embolic stroke, device malposition/thrombus, device embolization, airway event, initiation of dialysis, or other major AE).^21^ Additional intervention-specific outcomes were also recorded (Table 1).

### Statistical analysis

The ultimate goal of the current study was to evaluate whether there were disparities in referral, outcomes, and access to high-volume centers for pediatrric/congenital catheterization procedures. First, we sought to evaluate whether there were significant differences in distribution of race/ethnicity in procedures, so the proportion of each group was compared between case types. We also sought to determine if there were differences in the characteristics of subjects within case types that suggested that there was delay in reaching the catheterization laboratory. For instance, in shunt lesions (ASD and PDA closure), we evaluated whether the ratio of pulmonary to systemic output was higher in patients of different race-ethnicity (Table 1 details the case-type specific outcomes). Where available we sought to determine if the conduct of cases was similar between these same groups. We hypothesized that differences in age at referral could reflect disparities in access or referral. To evaluate this, we first evaluated the observed differences in age at referral between race and ethnicity using the Kruskal-Wallis test. To adjust for measurable confounders, we then calculated multivariable linear regression models with age as the primary outcome, race-ethnicity as the primary exposure, and covariates that were identified prior to analysis based on previous literature and clinical experience that might influence timing of procedure.^23^, ^24^, ^25^ For dichotomous variables, missing data were assumed normal (ie, not present) to avoid bias introduced by case restriction. We anticipated that insurance payer and race would be correlated so this was not included in these models.^17^^,^^35^^,^^36^ No model refinement procedures were performed. We did not explore whether any forms of effect modification/interaction between variables. For specific procedures, we also evaluated whether race-ethnicity were associated with severity of disease (eg, heart failure or ventricular dysfunction as an indication for balloon valvuloplasty). For these, multivariable logistic regression was used to evaluate the association adjusting for preidentified measurable confounders, using the same covariates as in prior models.

Third, we compared the likelihoods of AE for each case type between patients of different race-ethnicity groups using chi-squared test. For events with sufficient events, multivariable logistic regression models were calculated for the outcome with race-ethnicity as the exposure with same covariates as used in the prior model.

Fourth, as an exploratory analysis of potential disparities in access to care, we compared the proportion of different race-ethnicity groups between centers of different annual procedural volume. Annualized total PCCL volume (averaged over the study period) for each center was used as the exposure.^21^^,^^32^, ^33^, ^34^ We compared these proportions using the chi-squared test. Because the identity and other characteristics of centers are not ascertainable in IMPACT adjustment for measurable confounding was not possible.

All analyses were performed using SAS v9.4 (SAS Institute). The threshold for statistical significance was set at *P* < 0.05. For all multivariable models, collinearity was evaluated, as were normality of residuals. We acknowledge that addressing the primary study question necessitated a large number of comparisons. To avoid erroneous conclusions due to these multiple comparisons, we have identified the primary outcomes of interest and sought, where possible to see consistency across comparisons. No additional penalization for multiple comparisons was performed.

## Results

### Study population

In total, 20,632 cases were studied (Table 2), of which the total cases ranged from 931 cases for BAV and 9,844 cases for PDA closure cases. There was a significant difference in the proportion of patients of each race-ethnicity group between different procedures (*P* < 0.001). The proportion of non-Hispanic White patients undergoing BAV (72%; 95% CI: 69%-75%) was higher than those undergoing other procedures (52% to 60%) with a proportional decrease in the proportion of non-Hispanic Black patients (2%; 95% CI: 1%-3% compared to 10% to 16% for the other 3 procedures).

**Table 2:** Racial and Ethnic Composition of IMPACT Cases 7/1/2016-12/31/2022

	Non-Hispanic White	Hispanic	Non-Hispanic Black	Asian	Other
Device closure of atrial septal defect (n = 6,979)	57% (3,994) 95% CI: 56%-58%	25% (1,716) 95% CI: 24%-26%	10% (704) 95% CI: 9%-11%	6% (413) 95% CI: 5%-6%	2% (152) 95% CI: 2%-3%
Device closure of patent ductus arteriosus (n = 9,844)	52% (5,071) 95% CI: 52%-53%	25% (2,432) 95% CI: 24%-26%	16% (1,577) 95% CI: 15%-17%	5% (529) 95% CI: 5%-6%	2% (235) 95% CI: 2%-3%
Non-neonatal balloon aortic valvuloplasty (n = 931)	72% (671) 95% CI: 69%-75%	20% (182) 95% CI: 17%-22%	2% (21) 95% CI: 1%-3%	4% (34) 95% CI: 3%-5%	2% (23) 95% CI: 2%-4%
Non-neonatal balloon pulmonary valvuloplasty (n = 2,878)	60% (1,727) 95% CI: 58%-62%	20% (600) 95% CI: 19%-22%	13% (385) 95% CI: 12%-15%	4% (108) 95% CI: 3%-5%	2% (58) 95% CI: 2%-3%

Across all case types, the proportion of non-Hispanic White and Asian patients with commercial insurance was higher than that in Hispanic and non-Hispanic Black patients (Table 3, Table 4, Table 5, Table 6). Distance from the patient’s home address to the treating center was also higher for White and other-race patients compared to Hispanic, Black, and Asian patients (Table 3, Table 4, Table 5, Table 6).

**Table 3:** Study Population of Patients Undergoing Device Closure of Atrial Septal Defects (N = 6,979)

	Non-Hispanic White (n = 3,994)	Hispanic (n = 1,716)	Non-Hispanic Black (n = 704)	Non-Hispanic Asian (n = 413)	Other (n = 152)	*P* Value
Age, y	6 (IQR: 4-10)	6 (IQR: 4-11)	6 (IQR: 4-11)	7 (IQR: 4-12)	6 (IQR: 4-10)	0.01
Female	64% (2,556)	66% (1,131)	61% (429)	57% (2,236)	72% (109)	0.003
Height, cm	117 (IQR: 104-143)	118 (IQR: 104-146)	121 (IQR: 106-150)	120 (IQR: 105-150)	115 (IQR: 102-142)	0.001
Weight, kg	22 (IQR: 17-38)	23 (IQR: 17-42)	24 (IQR: 17-47)	21 (IQR: 16-44)	23 (IQR: 16-40)	<0.001
BMI, kg/m^2^	16 (IQR: 15-19)	17 (IQR: 15-20)	17 (IQR: 15-20)	16 (IQR: 14-19)	17 (IQR: 15-19)	<0.001
Insurance						
Commercial	52% (2,061)	25% (437)	29% (201)	57% (237)	34% (52)	<0.001
Public	27% (1,091)	55% (945)	63% (443)	29% (121)	51% (78)	
Other/Missing	21% (842)	19% (334)	9% (60)	13% (55)	14% (22)	
Chronic lung disease	1.5% (61)	1.3% (22)	2.3% (16)	1.5% (6)	4.0% (6)	0.07
Heart failure	0.5% (21)	1.5% (25)	0.3% (2)	1.0% (4)	1.3% (2)	0.002
History of arrhythmia	1.5% (60)	1.4% (24)	2.7% (19)	1.5% (6)	0.7% (1)	0.13
Hemodynamic vulnerability						
Systemic arterial saturation<95%	6% (238)	9% (156)	4% (28)	6% (24)	7% (10)	<0.001
Mixed venous saturation<60%	0.6% (24)	0.8% (13)	0.7% (5)	0.5% (2)	0.7% (1)	0.95
Systemic ventricular end-diastolic pressure >18 mm Hg	0.2% (9)	0.2% (3)	0.1% (1)	0.7% (3)	0% (0)	0.32
Main pulmonary artery systolic pressure >45 mm Hg	0% (0)	0.1% (2)	0% (0)	0% (0)	0% (0)	0.19
PVRi >2.8 WU/m^2^	1.3% (52)	2.6% (44)	1.4% (10)	1.5% (60)	2.0% (3)	0.02
Qp:Qs ratio>1.2	70% (2,791)	77% (1,327)	74% (524)	81% (336)	78% (119)	<0.001
Qp:Qs ratio	1.6 (IQR: 1.3-2.1)	1.7 (IQR: 1.4-2.1)	1.7 (IQR: 1.4-2.1)	1.8 (IQR: 1.5-2.4)	1.8 (IQR: 1.5-2.3)	<0.001
Multifenestrated	17% (662)	15% (257)	16% (110)	10% (40)	16% (25)	0.006
Balloon sizing performed	75% (3,001)	75% (1,294)	79% (557)	80% (330)	70% (106)	0.02
Rim measurements performed	66% (2,635)	73% (1,245)	71% (499)	69% (284)	71% (108)	<0.001
Any deficient rim (see below)	9% (280)	10% (165)	7% (39)	10% (42)	9% (13)	0.26
Distance to treating center (miles)	37 (IQR: 16-86) n = 1,885	19 (IQR: 9-55) n = 886	16 (IQR: 6-47) n = 366	14 (IQR: 8-26) n = 167	21 (IQR: 11-151) n = 63	<0.001

Values are median (IQR) or % (n).

BMI = body mass index; PVRI = indexed pulmonary vascular resistance; Qp:Qs = ratio of pulmonary to systemic blood flow.

**Table 4:** Study Population of Patients Undergoing Device Closure of Patent Ductus Arteriosus (N = 9,844)

	Non-Hispanic White (n = 5,071)	Hispanic (n = 2,432)	Non-Hispanic Black (n = 1,577)	Non-Hispanic Asian (n = 529)	Other (n = 235)	*P* Value
Age (y)	2 IQR: 1-5	3 IQR: 1-5	2 IQR: 1-5	3 IQR: 2-5	2 IQR: 1-4	<0.001
Female	63% (3,211)	64% (1,565)	62% (979)	70% (370)	66% (155)	0.02
Height (cm)	88 IQR: 74-107	86 IQR: 72-106	83 IQR: 71-104	88 IQR: 73-104	84 IQR: 75-104	0.001
Weight (kg)	13 IQR: 9-18	12 IQR: 9-18	12 IQR: 8-17	12 IQR: 8-17	12 IQR: 9-17	<0.001
BMI (kg/m^2^)	16 IQR: 15-18	16 IQR: 15-18	16 IQR: 15-18	16 IQR: 14-17	16 IQR: 15-17	<0.001
Insurance						<0.001
Commercial	47% (2,381)	25% (596)	28% (441)	57% (299)	30% (70)	
Medicaid	34% (1,719)	59% (1,435)	62% (978)	29% (153)	55% (132)	
Other	19% (971)	16% (401)	10% (158)	15% (77)	14% (33)	
Indication						<0.001
Spontaneous bacterial endocarditis prophylaxis	39% (1,982)	33% (808)	34% (529)	32% (168)	34% (80)	
Left ventricular volume overload	61% (3,089)	67% (1,624)	66% (1,048)	68% (361)	66% (155)	
Chronic lung disease	4% (217)	5% (133)	9% (146)	6% (31)	6% (13)	<0.001
Heart failure	1% (73)	3% (72)	3% (43)	4% (20)	3% (7)	<0.001
Arrhythmia	1% (55)	1% (34)	1% (23)	1% (5)	0.4% (1)	0.45
Hemodynamic vulnerability						
Systemic arterial saturation <95%	12% (556/3,788)	12% (359/2,323)	17% (249/1,465)	15% (75/497)	16% (36/229)	<0.001
Mixed venous saturation <60%	3% (143/4,328)	5% (102/2,199)	6% (77/1,358)	5% (25/466)	2% (5/211)	<0.001
Systemic ventricular end-diastolic pressure>18 mm Hg	3% (100/3,649)	3% (48/1,790)	3% (36/1,146)	5% (19/371)	3% (5/182)	0.12
PVRi >2.8 WU/m^2^	3% (110/2,972)	4% (74/1,945)	4% (51/1,168)	5% (19/408)	4% (7/178)	0.12
Qp:Qs ratio >1.2	53% (2,024/3,852)	58% (1,168/2,029)	60% (719/1,201)	64% (270/420)	57% (104/182)	<0.001
Qp:Qs ratio (missing)	1.3 IQR: 1.0-1.7 (1,219)	1.3 IQR: 1.1-1.8 (403)	1.4 IQR: 1.1-1.9 (376)	1.4 (1.1-2.0) (109)	1.3 (1.1-1.7) (53)	<0.001
PDA diameter aortic side (mm) (missing)	7 IQR: 5-9 (103)	8.0 IQR: 6-10 (34)	7 IQR 5-10 (35)	8 IQR: 6-10 (11)	8 IQR: 6-10 3	<0.001
PDA minimum diameter (mm) (missing)	2 IQR: 2-3 (64)	2 IQR: 2-3 (16)	2 IQR: 2-3 (18)	2 IQR: 2-3 (8)	2 IQR 2-3 (1)	<0.001
PDA length (mm) (missing)	9 IQR: 7-12 (157)	9 IQR: 7-12 (79)	10 IQR: 8-14 (63)	9 IQR: 7-11 (22)	9 IQR: 7-12 (3)	<0.001
PDA classification						<0.001
Type A (conical)	63% (3,208)	66% (1,615)	51% (800)	70% (372)	71% (168)	
Type B (window)	2% (79)	1% (35)	1% (22)	2% (11)	2% (5)	
Type C (tubular)	14% (708)	14% (335)	22% (346)	15% (81)	11% (27)	
Type D (complex)	5% (259)	5% (114)	6% (90)	2% (12)	2% (4)	
Type E (elongated)	15% (738)	13% (306)	19% (296)	9% (46)	12% (28)	
Missing	2% (79)	1% (27)	1% (23)	1% (7)	1% (3)	
Distance to treating center (miles)	40 (IQR: 17-90)	19 (IQR: 9-53)	18 (IQR: 8-58)	16 (IQR: 8-32)	33 (IQR: 12-89)	<0.001

Values are median (IQR) or % (n).

PDA = patent ductus arteriosus; other abbreviations as in Table 3.

**Table 5:** Characteristics of Patients Undergoing Balloon Pulmonary Valvuloplasty (N = 2,878)

	Non-Hispanic White (n = 1,727)	Hispanic (n = 600)	Non-Hispanic Black (n = 385)	Asian (n = 108)	Other (n = 58)	*P* Value
Age (y)	0 IQR: 0-1	0 IQR 0-2	0 IQR: 0-1	0 IQR: 0-3	0 IQR: 0-2	0.07
Female	51% (881)	53% (315)	50% (191)	49% (53)	53% (31)	0.89
Height (cm)	61 IQR: 55-77	63 IQR: 55-84	63 IQR: 54-76	63 IQR: 55-99	64 IQR: 55-91	0.32
Weight (kg)	6.2 IQR: 4.6-10.3	6.9 IQR: 4.7-12.4	6.6 IQR: 4.6-10.3	6.6 IQR: 4.9-16.7	7.1 IQR: 4.8-13.5	0.09
BMI (kg/m^2^)	16 IQR: 15-18	16 IQR: 15-18	16 IQR: 15-18	17 IQR: 14-18	16 IQR: 14-18	0.23
Insurance						<0.001
Commercial	46% (795)	25% (151)	23% (87)	50% (54)	35% (20)	
Medicaid	34% (590)	60% (360)	67% (259)	37% (40)	48% (28)	
Other	20% (342)	15% (89)	10% (39)	13% (14)	17% (10)	
Any genetic syndrome	% (n)					
Chronic lung disease	5% (78)	8% (48)	9% (35)	2% (2)	3% (2)	<0.001
Heart failure	1% (17)	2% (14)	2% (6)	2% (2)	3% (2)	0.10
Arrhythmia	2% (27)	2% (11)	1% (3)	4% (4)	2% (1)	0.30
Hemodynamic vulnerability						
Systemic arterial saturation<95%	28% (433/1,508)	29% (163/554)	28% (95/342)	27% (28/102)	26% (14/53)	0.97
Mixed venous saturation<60%	12% (176/1,506)	12% (69/567)	13% (48/359)	6% (6/102)	6% (3/52)	0.20
Systemic ventricular end-diastolic pressure>18 mm Hg	1% (8/595)	1% (3/242)	3% (4/150)	2% (1/108)	0% (0/19)	0.72
PVRi >2.8 WU∗m^2^	6% (56/1,014)	7% (28/414)	10% (25/252)	7% (5/72)	8%(3/37)	0.17
Qp:Qs ratio>1.2	12% (145/1,222)	17% (79/466)	16% (45/291)	18% (14/80)	15% (6/41)	0.05
Indication						0.05
High resting gradient	96% (1,666)	93% (558)	95% (366)	90% (97)	98% (57)	
RV dysfunction	4% (61)	7% (42)	5% (5)	10% (11)	2% (1)	
Dysplastic pulmonary valve	51% (883)	54% (322)	55% (210)	41% (44)	53% (31)	0.10
Preintervention gradient measured	94% (1,616)	94% (563)	91% (352)	93% (100)	88% (51)	0.24
Preintervention gradient	41 IQR: 32-54	44 IQR: 33-56	42 IQR: 33-54	40 IQR: 31-53	50 IRQ: 35-73	0.01
Distance from home to center (miles)	31 IQR: 14-76 Missing: 959	21 IQR: 9-60 Missing: 238	16 IQR: 6-46 Missing: 198	16 IQR: 7-25 Missing: 60	45 IQR: 9-110 Missing: 30	<0.0001

Values are median (IQR) or % (n).

RV = right ventricular and other abbreviations as in Table 3.

**Table 6:** Characteristics of Patients Undergoing Balloon Aortic Valvuloplasty (N = 931)

	Non-Hispanic White (n = 671)	Hispanic (n = 182)	Non-Hispanic Black (n = 21)	Asian (n = 34)	Other (n = 23)	*P* Value
Age (y)	1 IQR: 0-10	1 IQR 0-11	1 IQR: 0-12	0 IQR: 0-2	0 IQR: 0-9	0.23
Female	26% (171)	25% (45)	62% (13)	27% (9)	26% (6)	0.006
Height (cm)	68 IQR: 56-141	76 IQR: 57-146	85 IQR: 60-152	67 IQR: 54-90	66 IQR: 57-122	0.23
Weight (kg)	8.0 IQR: 4.9-41.9	10.0 IQR: 5.1-46.2	11.0 IQR: 5.91-42.2	7.9 IQR: 4.4-14.6	7.5 IQR: 5.4-22.5	0.23
BMI (kg/m^2^)	17 IQR: 15-20	17 IQR: 15-22	16 IQR: 15-20	16 IQR: 15-18	17 IQR: 15-22	0.13
Insurance						<0.001
Commercial	51% (345)	31% (56)	24% (5)	71% (24)	39% (9)	
Medicaid	29% (193)	54% (98)	57% (12)	26% (9)	48% (11)	
Other	20% (133)	15% (28)	19% (4)	3% (1)	13% (3)	
Chronic lung disease	2% (13)	4% (7)	10% (2)	6% (2)	0% (0)	0.08
Heart failure	2% (13)	5% (9)	5% (1)	0% (0)	0% (0)	0.13
Arrhythmia	2% (10)	1% (2)	5% (1)	0% (0)	0% (0)	0.59
Hemodynamic vulnerability						
Systemic arterial saturation <95%	21% (127/616)	22% (39/174)	39% (7/18)	28% (8/29)	25% (5/20)	0.36
Mixed venous saturation <60%	6% (31/520)	8% (11/145)	0% (0/19)	17% (4/23)	0% (0/15)	0.11
Systemic ventricular end-diastolic pressure>18 mm Hg	17% (105/619)	21% (35/169)	16% (3/19)	22% (7/32)	0% (0/21)	0.18
Indication						0.001
High resting gradient	90% (603)	78% (142)	90% (19)	85%% (29)	87% (20)	
LV dysfunction or symptoms	10% (68)	22% (40)	10% (2)	15% (5)	13% (3)	
Bicuspid aortic valve	81% (541)	72% (131)	76% (16)	71% (24)	70% (16)	0.06
Preprocedure gradient measured	100% (671)	100% (182)	100% (21)	100% (34)	100% (23)	1.00
Preprocedure gradient	53 IQR: 44-66	55 IQR: 45-70	50 IQR: 42-62	55 IQR: 45-64	55 IQR: 44-70	0.59
Aortic valve diameter	10.4 IQR: 9-18 (n = 666)	11.2 IQR: 8.6-18.0 (n = 182)	10.1 IQR: 8.7-21.0 (n = 19)	10.0 IQR: 8.0-12.9 (n = 34)	9.5 IQR: 9.0-16.0 (n = 23)	0.15
Preprocedure insufficiency						0.01
None	68% (455)	68% (124)	57% (12)	74% (25)	65% (15)	
1+	25% (170)	25% (46)	19% (4)	21% (7)	26% (6)	
2+	4% (26)	3% (5)	5% (1)	3% (1)	4% (1)	
3+	1% (6)	2% (3)	14% (3)	3% (1)	0% (0)	
4+	2% (14)	2% (4)	5% (1)	0% (0)	4% (1)	
Distance from home to center (miles)	37 IQR: 19-88 Missing: 367	19 IQR: 10-94 Missing: 87	14 IQR: 7-43 Missing: 11	16 IQR: 5-36 Missing: 22	22 IQR: 6-67 Missing: 12	0.002

Values are median (IQR) or % (n).

LV = left ventricular.

### Device closure of atrial septal defects

In total, 6,979 cases were studied (Supplemental Figure 1) and the characteristics of the study population are described in Table 3. ASD closure was performed at an older age in Asian patients (median 7: IQR: 4-12) compared to other 4 groups (*P* = 0.01). Qp:Qs ratio was also significantly higher in patients with Asian and other-race patients (*P* < 0.001). The proportion of patients described as being in heart failure was 0.5% in White patients but significantly higher in Hispanic (1.5%), Asian (1%), and other race (1.3%) patients (*P* = 0.002). Statistically significant differences were noted in patient sex, height, weight, BMI, and the proportion of patients with low systemic arterial saturation between race-ethnicity groups, but whether these were clinically meaningful was not clear (Table 3).

In analyses adjusted for measurable confounders, age of ASD closure was higher in Hispanic (*P* = 0.007), Black (*P* = 0.02), and Asian (*P* = 0.001) patients than in White patients (Supplemental Table 1). In these models, larger Qp:Qs, deficient rim, low arterial saturation, low mixed venous saturation, and chronic lung disease were all also associated with device closure at a younger age (Supplemental Table 1).

In terms of outcomes, there were no significant differences in the likelihoods of death prior to discharge, catastrophic AE, or major AE (Table 7). In terms of measures of technical success, residual shunts were more common in Black patients (4%) than in other groups (*P* = 0.03). There was no statistically significant difference in the risk of device embolization between the groups (*P* = 0.30). Adjusted analyses did not demonstrate significant differences in the likelihood of AE between race-ethnicity groups (data not shown).

**Table 7:** Adverse Events After Device Closure of Atrial Septal Defects

	Non-Hispanic White (n = 3,994)	Hispanic (n = 1,716)	Non-Hispanic Black (n = 704)	Asian (n = 413)	Other (n = 152)	*P* Value
Death prior to discharge	0.03% (1)	0.1% (1)	0.1% (1)	0% (0)	0% (0)	0.69
Catastrophic adverse event	0.9% (37)	0.8% (14)	0.7% (5)	1.5% (6)	2.0% (3)	0.46
Major adverse event	2% (99)	3% (46)	2% (16)	3% (11)	5% (7)	0.69
More than trivial residual shunt	2% (86)	3% (49)	4% (29)	3% (11)	2% (3)	0.03
Device embolization	0.8% (33)	1.1% (19)	0.6% (4)	1.7% (7)	1.3% (2)	0.30

Values are % (n).

### Device closure of patent ductus arteriosus

In total, 9,844 PDA closure cases were studied (Supplemental Figure 2), the characteristics of which are described in Table 4. Between race-ethnicity groups, the age at PDA closure was older in Hispanic and Asian patients (*P* < 0.001), with higher Qp:Qs (*P* < 0.001). Asian patients had reported heart failure (4%) symptoms more often than white patients (1%, *P* < 0.001). The proportion of patients undergoing PDA closure of spontaneous bacterial endocarditis prophylaxis was significantly higher in non-Hispanic White patients (39%) than the other 4 groups (*P* < 0.001). In terms of anatomic classification, Black patients had a higher proportion of type C (22%) and type E (9%) PDA than the other groups with a proportional decrease in type A PDA (*P* < 0.001).

In analyses adjusted for measurable confounders, the point estimate for the association between Hispanic ethnicity (beta = 59) and Asian race (beta = 91) and age at PDA closure were consistent with older age but the associations were not significant (*P* = 0.13 and 0.20), respectively. In this model, high Qp:Qs, low systemic arterial saturation, low mixed venous saturation, and left ventricular volume overload as the indication were all associated with younger age of PDA closure while minimal diameter of the PDA<2 mm was associated with older age at PDA closure (Supplemental Table 2).

Recognizing that the subgroups of patients undergoing device closure of PDA for the indication of left ventricular volume overload and endocarditis prophylaxis might be qualitatively different in a way that could affect the association between race and age of closure, the characteristics of individuals undergoing PDA closure were compared by indication as a sensitivity analysis. Left ventricular volume overload as an indication was associated with younger age at closure (median 1 IQR: 1-3 vs median 3: IQR: 2-6; *P* < 0.001, Supplemental Table 3). The indication of volume overload was also associated with a higher likelihood of report of heart failure, chronic lung disease, low systemic arterial saturation, low mixed venous saturation, high systemic ventricular end-diastolic pressure, high indexed pulmonary vascular resistance, and high Qp:Qs as well as a higher likelihood of a type A PDA. A higher proportion of patients undergoing PDA closure for endocarditis prophylaxis were non-Hispanic White (56% vs 49%; *P* < 0.001). Analyzing the association between race-ethnicity and age at PDA closure restricted to either indication did not change the associations described in the original model (Supplemental Tables 4 and 5).

In terms of AE, there were no differences in the likelihood of death prior to discharge between different race-ethnicity groups (Table 8). However, the likelihood of catastrophic AE was higher in Black and Asian patients than in White patients (*P* = 0.01). The likelihood of major AE was higher in Hispanic, Black, Asian, and other race patients than White patients (*P* = 0.001). In terms of technical outcomes, the likelihood of nontrivial residual shunt (*P* = 0.002), device embolization (*P* = 0.05), and pulmonary artery obstruction (*P* = 0.02) were all higher in Asian and Black patients than White patients.

**Table 8:** Adverse Outcomes After Device Closure of Patent Ductus Arteriosus

	Non-Hispanic White (n = 5,071)	Hispanic (n = 2,432)	Non-Hispanic Black (n = 1,577)	Non-Hispanic Asian (n = 529)	Other (n = 235)	*P* Value
Death prior to discharge	0.1% (7)	0.1% (3)	0.4% (6)	0.4% (2)	0% (0)	0.20
Catastrophic adverse event	1% (47)	1% (23)	2% (27)	2% (11)	0.4% (1)	0.01
Major adverse event	1%(73)	2% (42)	2% (39)	4% (19)	2% (4)	0.001
Residual shunt	0.9% (46)	1% (24)	2% (25)	3%(14)	0.9% (2)	0.002
Device embolization	1% (73)	1% (34)	2% (37)	2% (9)	0.4% (1)	0.05
Aortic obstruction	0.1% (23)	0.5% (12)	0.4% (7)	0.4% (2)	0.4% (1)	1.00
Pulmonary artery obstruction	0.03% (14)	0.2% (14)	0.8% (13)	0.9% (5)	0.4% (1)	0.02

Values are % (n).

After adjusting for measurable confounders, Black (OR: 1.3) and Asian race (OR: 1.9) had point estimates consistent with higher risk of composite major AE but the associations were no longer significant (*P* = 0.27 and 0.07, respectively) (Supplemental Table 6). Further analyses of other events were not performed because the number of events rates was prohibitively low.

### Balloon pulmonary valvuloplasty

In total, 2,878 cases were studied (Supplemental Figure 3). The characteristics of the patients and procedures are depicted in Table 5. No significant differences were seen in the age at BPV (*P* = 0.07) between different race-ethnicity groups. Similarly, there were no significant differences in the proportion of cases described as experiencing heart failure (*P* = 0.10). The proportion of cases with right ventricular (RV) dysfunction as the indication for intervention was higher in Hispanic (7%), Black (5%), and Asian (10%) patients than in White ones (4%, *P =* 0.05). A significant association between race-ethnicity and preintervention gradient was identified (*P* = 0.01), but the notable difference was that other race patients had a higher gradient than other groups. In terms of other potential covariates, the proportion of Hispanic and Black patients with chronic lung disease was significantly higher than in other race-ethnicity groups (*P* < 0.001).

After adjusting for hemodynamic vulnerability, right ventricular dysfunction, and dysplastic pulmonary valve, Asian race was associated with greater age at BPV (beta: 283, *P* = 0.02) (Supplemental Table 7). A second model was calculated to determine if race-ethnicity were associated with the likelihood of the compositive outcome of heart failure and/or RV dysfunction. In this analysis Hispanic (OR: 1.6, *P* = 0.02) and Asian (OR: 3.2, *P* = 0.0008) race-ethnicity were associated with increased odds of the composite outcome relative to white patients (Supplemental Table 8).

In terms of outcomes, no significant association was found between race-ethnicity and the likelihood of death or major adverse outcomes (Table 9). The risk of catastrophic adverse outcomes was higher in Hispanic (3.6%) and Asian (2.8%) patients relative to white patients (1.5% *P* = 0.04). In terms of technical success, Hispanic and Asian race-ethnicity were associated with both a higher postprocedure gradient (*P* < 0.001) and a higher likelihood of a postprocedure gradient >50 mm Hg (*P* = 0.004).

**Table 9:** Adverse Outcomes After Balloon Pulmonary Valvuloplasty

	Non-Hispanic White (n = 1,727)	Hispanic (n = 385)	Non-Hispanic Black (n = 600)	Non-Hispanic Asian (n = 108)	Other (n = 58)	*P* Value
Death prior to discharge	0.8% (14)	1.6% (5)	0.8% (5)	0.9% (1)	0% (0)	0.63
Catastrophic adverse event	1.5% (26)	3.6% (14)	1.5% (9)	2.8% (3)	0% (0)	0.04
Major adverse event	2.8% (48)	4.2% (16)	2.7% (16)	3.7% (4)	1.7% (1)	0.59
Postprocedure gradient	15 IQR: 10-23	18 IQR: 10-22	17 IQR: 12-25	14 IQR: 10-22	19 IQR: 12-35	<0.001
Postprocedure gradient>50 mm Hg	3% (44)	4% (14)	6% (34)	2% (2)	6% (3)	0.004
Balloon rupture	4% (76)	3% (12)	4% (26)	2% (2)	2% (1)	0.45

Values are median (IQR) or % (n).

After adjusting for hemodynamic vulnerabilities (systemic arterial saturation and mixed venous saturation) and typical vs dysplastic pulmonary valve, the observed association between race-ethnicity and catastrophic AE was no longer observed (Supplemental Table 9).

### Balloon aortic valvuloplasty

In total, 931 BAV cases were studied (Supplemental Figure 4). There was no significant association between race-ethnicity and age at BAV (*P* = 0.23). However, Hispanic and Asian race-ethnicity were associated with higher likelihood of LV dysfunction (22% and 15%) compared to White patients (10%, *P* = 0.001). Black patients were more likely to have aortic insufficiency before intervention (43% vs 32% in White and Hispanic patients, *P* = 0.01). There was not a significant association between race-ethnicity and other confounders (Table 6). In a model adjusting for low systemic arterial saturation and bicuspid aortic valve, Asian race-ethnicity was associated with younger age at BAV (beta: −762, *P* = 0.04) (Supplemental Table 10). No other associations between race-ethnicity and age at BAV were seen. In a model evaluating the likelihood of experiencing either heart failure or LV dysfunction adjusted for the same confounders, however, Hispanic race-ethnicity was associated with increased likelihood (OR: 2.6; *P* = 0.0005) relative to White race-ethnicity (Supplemental Table 11).

In terms of adverse outcomes, Black (10%), Hispanic (2%), and Asian (3%) race-ethnicity were all associated with a higher likelihood of death before discharge (10%) than White patients (1%, *P* = 0.001) (Table 10). Significant associations were not seen between catastrophic and pooled major AE and race-ethnicity (*P =* 0.05 and 0.12, respectively). Race-ethnicity was associated with the postprocedure gradient with higher gradients seen in Black, Asian, and other race patients (*P* = 0.007). The likelihood of a residual gradient >50 mm Hg was not significantly associated with race-ethnicity (*P* = 0.11) though the point estimates for Black, Asian, and other race patients were suggestive of higher likelihood. The risk of new moderate or worse aortic insufficiency after BAV was not significantly associated with race-ethnicity.

**Table 10:** Adverse Outcomes After Balloon Aortic Valvuloplasty

	Non-Hispanic White (n = 671)	Hispanic (n = 182)	Non-Hispanic Black (n = 21)	Asian (n = 34)	Other (n = 23)	*P* Value
Death prior to discharge	1% (4)	2% (4)	10% (2)	3% (1)	0% (0)	0.001
Catastrophic adverse event	5% (32)	5% (9)	14% (3)	15% (5)	4% (1)	0.05
Major adverse event	7% (45)	8% (15)	19% (4)	15% (5)	9% (2)	0.12
Postprocedure peak gradient	20 (IQR: 15-28)	20 (IQR: 14-29)	25 (IQR: 17-33)	26 (IQR: 17-34)	25 (IQR: 19-34)	0.007
Postprocedure peak gradient >50 mm Hg	2% (13)	2% (3)	5% (1)	6% (2)	9% (2)	0.11
Post procedure AI ≥moderate	13% (87)	10% (19)	24% (5)	18% (6)	13% (3)	0.37
Balloon rupture	5% (36)	5% (10)	10% (92)	12% (4)	4% (1)	0.54

Values are median (IQR) or % (n).

In a model adjusting for LV dysfunction, low systemic arterial saturation, and bicuspid aortic valve Asian race-ethnicity (OR: 3.2; *P* = 0.03) was associated with increased odds of catastrophic AE relative to White patients (Supplemental Table 12). In the same model, the point estimate for the association between Black race-ethnicity and catastrophic AE was suggestive of higher likelihood (OR: 3.1) but the association was not significant (*P* = 0.08). In a model for composite major AE, Asian (OR: 2.3; *P* = 0.11) and Black (OR: 3.2; *P* = 0.05) race-ethnicity had point estimates suggestive of an association with increased risk, but the association was not statistically significant (Supplemental Table 13).

### Association between center annual volume and distribution of race-ethnicity

As an exploratory analysis to evaluate potential disparities in access, the distribution of patient race-ethnicity and the annual catheterization laboratory case volume (divided into quartiles) was performed. For all 4 procedures analyzed, there was a significant association between race-ethnicity and program volume (Table 11, 12, 13, 14). For all procedures, the proportion of Hispanic patients was highest in the lowest-volume quartile centers and was lower at higher volume centers. For ASD cases, the proportion of Hispanic patients at the lowest volume centers was 33% while it was 25% at the highest volume centers. Smaller differences were seen for Black patients undergoing device closure of ASD (12% at lowest volume centers and 9% at highest volume centers) and BPV (15% at the lowest volume centers and 11% at the highest volume centers). These differences were reflected by higher proportions of non-Hispanic White patients at the highest volume centers compared to lower volume centers. We acknowledge that there are not clear trends across all strata for each procedure.

**Table 11:** Association of Center Annual Procedural Volume and Race-Ethnicity of Patients Undergoing ASD Closure (N = 6,979)

N = 6,979	Non-Hispanic White (n = 3,994)	Hispanic (n = 1,716)	Non-Hispanic Black (n = 704)	Asian (n = 413)	Other (n = 152)	*P* Value
Volume quartile 1	44% (199)	33% (148)	12% (54)	7% (31)	4% (18)	<0.001
Volume quartile 2	54% (594)	26% (287)	12% (136)	6% (65)	2% (20)	
Volume quartile 3	62% (1,113)	21% (375)	10% (186)	6% (100)	2% (27)	
Volume quartile 4	58% (2,088)	25% (906)	9% (328)	6% (217)	2% (87)	

ASD = atrial septal defect.

Values are % (n). Quartile 1 has the lowest volume, and quartile 5 has the highest volume.

**Table 12:** Association of Center Annual Procedural Volume and Race-Ethnicity of Patients Undergoing PDA Closure (N = 9,844)

	Non-Hispanic White (n = 5,071)	Hispanic (n = 2,432)	Non-Hispanic Black (n = 1,577)	Asian (n = 529)	Other (n = 235)	*P* Value
Volume quartile 1	45% (349)	30% (229)	15% (113)	8% (58)	3% (21)	<0.001
Volume quartile 2	49% (798)	25% (413)	17% (280)	6% (101)	2% (34)	
Volume quartile 3	54% (1,422)	22% (584)	18% (484)	4% (94)	2% (64)	
Volume quartile 4	52% (2,502)	25% (1,206)	15% (700)	6% (276)	2% (116)	

Abbreviation as in Table 4.

Values are % (n). Quartile 1 has the lowest volume, and quartile 5 has the highest volume.

**Table 13:** Association of Center Annual Procedural Volume and Race-Ethnicity of Patients Undergoing Balloon Pulmonary Valvuloplasty (N = 2,878)

	Non-Hispanic White (n = 1,727)	Hispanic (n = 600)	Non-Hispanic Black (n = 385)	Asian (n = 108)	Other (n = 58)	*P* Value
Volume quartile 1	45% (87)	30% (58)	15% (29)	6% (12)	4% (8)	<0.001
Volume quartile 2	60% (304)	22% (111)	13% (64)	4% (21)	1% (5)	
Volume quartile 3	59% (469)	20% (155)	17% (134)	3% (22)	2% (15)	
Volume quartile 4	63% (867)	20% (276)	11% (158)	4% (53)	2% (30)	

Values are % (n). Quartile 1 has the lowest volume, and quartile 5 has the highest volume.

**Table 14:** Association of Center Annual Procedural Volume and Race-Ethnicity of Patients Undergoing Balloon Aortic Valvuloplasty (N = 931)

	Non-Hispanic White (n = 671)	Hispanic (n = 182)	Non-Hispanic Black (n = 21)	Asian (n = 34)	Other (n = 23)	*P* Value
Volume quartile 1	58% (34)	32% (19)	2% (1)	3% (2)	5% (3)	0.02
Volume quartile 2	71% (112)	20% (31)	3% (4)	3% (5)	3% (5)	
Volume quartile 3	79% (212)	13% (36)	3% (9)	2% (5)	2% (5)	
Volume quartile 4	70% (313)	21% (96)	2% (7)	5% (22)	2% (10)	

Values are % (n). Quartile 1 has the lowest volume, and quartile 5 has the highest volume.

## Discussion

In this retrospective multicenter cohort study of U.S. PCCL centers, we evaluated whether there were significant disparities in access to cardiac catheterization interventions, demonstrating that significant disparities in 4 core transcatheter interventions (Central Illustration B). Hispanic, Black and Asian patients underwent device closure of ASD later than White patients, even after adjusting for measurable covariates. In patients undergoing BPV and BAV, Hispanic ethnicity was associated with evidence of more severe disease at treatment. AE were rare and there were not statistically significant associations between race-ethnicity and likelihood of AE in PDA closure and BPV cases. There do, however, appear to be significantly worse outcomes in Asian and Black patients after BAV. We also demonstrated that center volume was associated with the proportion of race-ethnicity treated at each center, with the smallest volume centers treating a higher proportion of Hispanic patients compared to the highest volume of centers (with similar patterns for Black patients undergoing ASD closure and BPV).

In the face of consistent evidence that non-White children with CHD consistently have worse outcomes than their White counterparts,^3^^,^^4^^,^^12^, ^13^, ^14^, ^15^, ^16^ it is imperative to work toward identifying means of mitigating these disparities. The current research demonstrates that for this panel of PCCL interventions, racial-ethnic minority groups are reaching care later (either by age or the severity of illness). Whether this is due to delay in diagnosis or referral to catheterization services is not ascertainable in this design, but it should inform future research and quality improvement efforts. Evidence for disparities in access to care also are seen in analyses demonstrating that Hispanic (and to a lesser degree black patients) represent care disproportionately at smaller volume centers. While there continues to be debate about whether larger centers necessarily deliver superior outcomes,^21^^,^^32^^,^^33^ this pattern should raise concern about whether our current referral patterns contribute to disparities. This suggests that mitigation efforts should focus on the preprocedural care of these patients. Disparities in access to primary care, cardiology, or referral from cardiology to the catheterization laboratory are all potential avenues for these efforts.

Differentiating between disparities in diagnosis and referral is an important distinction. Recent studies have identified disparities by both race and socioeconomic status in fetal diagnosis of complicated congenital heart disease,^37^, ^38^, ^39^ but to our knowledge evaluation of postnatal screening and identification of heart disease and its association with race-ethnicity and other social determinants of health have not been studied. These disparities could bias birth registry data, so it is not possible to know if the reported differences in referral are due to differences in incidence. Quantifying the portion of the population with undiagnosed congenital heart disease is beyond the scope of this study. In future studies, evaluation of age at diagnosis and/or referral to cardiology might clarify where these issues arise.

It is important to acknowledge that race and ethnicity represent important but incomplete descriptors of the social determinants of health. Primary language, insurance, present socioeconomic resources, and health care literacy are all potential contributing factors, along with the combined effects of exposure to generations of institutional racism. Evaluating these are all beyond the scope of this study, but it is important to acknowledge this and to consider incorporating these factors in future studies.

Distance between patient home address and outcomes is associated with both worse outcomes in the cardiac care of adults^40^^,^^41^ and there is concern that greater distance traveled may represent another disparity where the burden of long travel disproportionately affects already disadvantaged families. We were able to calculate distance from home zip code to the treating center in this data set, but did not see an association between distance and any of our outcomes. There are several limitations to home-to-center distance in our analysis. Distance traveled is an imperfect surrogate for access; it is an imperfect correlate to both the time needed to travel to the center and the burden that the distance imposes on families.

Finally, it is also important to acknowledge that race and ethnicity as reported in this study are those captured in the medical record and reported in IMPACT. Practices of how race-ethnicity data are captured in the electronic medical record are variable, and there is evidence that medical record reported race often differs from self-reported race, with under-reporting of Black and Hispanic in both adult and pediatric cohorts.^27^^,^^28^ The effect of inaccurate reporting of race and ethnicity is unpredictable, but misclassification likely would result in bias toward the null.

### Study Limitations

In addition to the limitations already discussed, we acknowledge that this retrospective cohort analysis was restricted to evaluating the data available in IMPACT and unmeasured confounding is possible. Though IMPACT is a large multicenter registry, there is the potential for type II error especially for rare events like catastrophic adverse outcomes. Evaluating disparities across a large registry maximizes external validity, it is possible that patients treated at different hospitals may experience different degrees of the disparities described. Evaluating this variation was beyond the scope of this study. Specific geographic regions, especially urban areas may be served by more than one PCCL center. While the demographic characteristics served by individual centers may differ, an advantage of the IMPACT Registry is that it reflects a large proportion of U.S. programs, so the overall demographics of the cohort are likely reflective of care within the United States. As cited in the Methods section, missing data about patient race-ethnicity were present in the registry. Because it was the primary exposure and likely not missing at random, case restriction was applied. The study period included the COVID-19 pandemic, which has had documented effects on patterns of referral to PCCL programs.^42^ Evaluating whether these aggravated observed disparities is beyond the scope of the current study. Finally, this study is ill-equipped to evaluate the cause of the observed disparities, which deserve attention, since they are important to incorporate in efforts to mitigate disparity.

## Conclusions

Despite these limitations, we conclude that the current research demonstrates measurable disparities in referral to PCCL procedures by race-ethnicity with non-White patients referred for intervention later and with a higher likelihood of severe disease. There is also evidence of differential access to the highest volume centers, with a higher proportion of non-Hispanic White patients at the highest volume centers. Though AE are rare, there is some signal that race-ethnicity were also associated with a higher risk of AE, though the associations were less consistent. Efforts to mitigate disparities in outcomes for these forms of CHD should focus on improved screening and access to the health care system including referral to subspecialists.

Perspectives **COMPETENCY IN PATIENT CARE 1:** There is evidence that patients belonging to racial and ethnic minority groups reach pediatric/congenital catheterization care later (reflected by older age or more severe disease).

**COMPETENCY IN PATIENT CARE 2:** The proportion of Black and Hispanic patients is lower at high-volume pediatric catheterization programs than at lower volume programs.

**TRANSLATIONAL OUTLOOK:** Though this study has inherent limitations, clinicians should consider that racial and ethnic minorities are subject to potential disparities in access with unclear etiology.

## Funding support and author disclosures

The research was supported by 10.13039/100005485American College of Cardiology Foundation. The IMPACT Research and Publications Committee reviewed the proposed application and the resulting manuscript for appropriateness but did not participate in the drafting of the manuscript. The views expressed are solely those of the authors, not reflecting funders and other supporting groups. The authors have reported that they have no relationships relevant to the contents of this paper to disclose.
