# Supplementary material for: Evaluating Racial Disparities in Access to Common Pediatric/Congenital Transcatheter Interventions
Source: JACC Adv. 2025 Sep 23;4(11):102158. doi: 10.1016/j.jacadv.2025.102158 (PMC12495325; doi:10.1016/j.jacadv.2025.102158)
Supplement: Supplemental Data [file mmc1.pdf]

	Beta	*95% CI*	*p*
Race/ethnicity			
Non-Hispanic white	1	n/a	n/a
Hispanic	124	33 to 214	0.007
Non-Hispanic black	153	25 to 280	0.02
Asian	266	105 to 427	0.001
Other	-43	-299 to 213	0.74
Qp:Qs>1.5	-222	-297 to -147	<0.0001
Any deficient rim	-175	-314 to -335	0.01
Hemodynamic vulnerability			
Low systemic arterial saturation	-161	-312 to -9	0.04
Low mixed venous saturation	-878	-1343 to -412	0.0002
Elevated systemic ventricular end-diastolic pressure	411	-366 to 1188	0.30
Elevated pulmonary artery mean pressure	-438	-2627 to 1752	0.70
Elevated pulmonary vascular resistance	474	180 to 768	0.002
Heart failure	-115	-539 to 310	0.60
Chronic lung disease	-647	-945 to -349	<0.0001

Supplementary Table 1: Multivariable model for age (days) of ASD closure

	Odds ratio	*95% CI*	*p*
Race/ethnicity			
Non-Hispanic white	1	n/a	n/a
Hispanic	59	-18 to 137	0.13
Non-Hispanic black	-20	-109 to 69	0.66
Asian	91	-49 to 231	0.20
Other	-210	-405 to -13	0.04
PDA anatomy			
Type A	1	n/a	n/a
Type C	-2	-94 to 91	0.97
Type B, D, or E	85	9.4 to 160	0.03
Hemodynamic vulnerability			
High Qp:Qs	-431	-550 to -312	<0.001
Low systemic arterial saturation	-412	-515 to -309	<0.001
Low mixed venous saturation	-419	-731 to -107	0.009
Elevated systemic ventricular end-diastolic pressure	-50	-284 to 185	0.68
Elevated pulmonary vascular resistance	-104	-324 to 116	0.36
Minimal diameter <2 mm	157	224 to 90	<0.001
LV volume overload vs. SBE prophylaxis	-582	-650 to -515	<0.001

Supplementary Table 2: Multivariable model for age at device closure of patent ductus arteriosus

N=9,844	**Spontaneous bacterial endocarditis prophylaxis (n=3,567)**	**Left ventricular volume overload (n=6,277)**	**P**
Age (years)	3 IQR: 2-6	1 IQR: 1-3	<0.001
Female sex	63% (2,254)	64% (4,025)	0.35
Height (cm)	98 IQR: 82-118	80 IQR: 69-99	<0.001
Weight (kg)	15 IQR: 11-23	11 IQR: 8-16	<0.001
BMI	16 IQR: 15-18	16 IQR: 15-17	<0.001
Race/Ethnicity			<0.001
Non-Hispanic white	56% (1,982)	49% (3,089)	
Hispanic	23% (808)	26% (1,624)	
Non-Hispanic black	15% (529)	17% (1048)	
Non-Hispanic Asian	5% (168)	6% (361)	
Other	2% (80)	2% (155)	
Insurance			0.007
Commercial	40% (1,427)	38% (2,360)	
Medicaid	43% (1,526)	46% (2,891)	
Other	17% (614)	16% (1,026)	
Chronic lung disease	5% (166)	6% (374)	0.006
Heart failure	1% (22)	3% (193)	<0.001
Arrhythmia	1% (47)	1% (71)	0.42
Hemodynamic vulnerability			
Low systemic arterial saturation (y/n)	10% (319/3,338)	16% (956/5,865)	<0.001
Low mixed venous saturation (y/n)	1% (29/2,986)	6% (323/5,576)	<0.001
High systemic ventricular end-diastolic pressure (y/n)	1% (36/2,452)	4% (172/4,580)	<0.001
High PVRi (y/n)	2% (63/2,563)	4% (198/4,785)	<0.001
High Qp:Qs (y/n)	29% (791/2,703)	70% (3,494/4,981)	<0.001
Qp:Qs ratio<br>(missing)	1.1 IQR: 1.0-1.3<br>(864)	1.5 IQR: 1.2-2.0<br>(1,296)	<0.001
PDA diameter aortic side (mm)<br>(missing)	7 IQR: 4-9<br>(91)	8.0 IQR: 6-10<br>(145)	<0.001
PDA minimum diameter (mm) (missing)	2 IQR: 1-2<br>(91)	3 IQR: 2-3<br>(78)	<0.001
PDA length (mm)<br>(missing)	9 IQR: 7-11<br>(171)	9 IQR: 7-12<br>(233)	<0.001
PDA classification			<0.001
Type A (conical)	61% (2,161)	64% (4,002)	
Type B (window)	1% (47)	2% (105)	
Type C (tubular)	13% (455)	17% (1,042)	
Type D (complex)	5% (195)	5% (284)	
Type E (elongated)	19% (666)	12% (748)	
Missing	1% (43)	2% (96)	
Distance to treating center (miles)	28 (IQR: 12-78)<br>Missing: 1,655	26 (IQR: 11-74)<br>Missing 3,191	0.17

Supplementary Table 3: Characteristics of PDA cases by indication

	Odds ratio	*95% CI*	*p*
Race/ethnicity			
Non-Hispanic white	1	n/a	n/a
Hispanic	82	-8 to 173	0.07
Non-Hispanic black	-110	-213 to -7	0.04
Asian	73	-86 to 232	0.37
Other	-169	-389 to 52	0.13
PDA anatomy			
Type A	1	n/a	n/a
Type C	-44	-149 to 62	0.41
Type B, D, or E	-8	-100 to 83	0.86
Hemodynamic vulnerability			
High Qp:Qs	-337	-450 to -223	<0.001
Low systemic arterial saturation	-396	-509 tp -284	<0.001
Low mixed venous saturation	-409	-711 to -108	0.008
Elevated systemic ventricular end-diastolic pressure	-1.1	-239 to 237	0.99
Elevated pulmonary vascular resistance	-14	-250 to 222	0.91
Minimal diameter <2 mm	-42	-149 to 62	0.42

Supplementary Table 4: Multivariable model for age at device closure of patent ductus arteriosus (indication of left ventricular volume overload only)

	Odds ratio	*95% CI*	*p*
Race/ethnicity			
Non-Hispanic white	1	n/a	n/a
Hispanic	30	-104 to 163	0.66
Non-Hispanic black	116	-38 to 270	0.14
Asian	136	-116 to 388	0.29
Other	-250	-606 to 107	0.17
PDA anatomy			
Type A	1	n/a	n/a
Type C	59	-106 to 225	0.48
Type B, D, or E	194	70 to 318	0.002
Hemodynamic vulnerability			
High Qp:Qs	-881	-1,240 to -521	<0.001
Low systemic arterial saturation	-451	-649 to -253	0.38
Low mixed venous saturation	-389	-1,266 to 487	0.38
Elevated systemic ventricular end-diastolic pressure	-141	-680 to 398	0.61
Elevated pulmonary vascular resistance	-268	-706 to 170	0.23
Minimal diameter <2 mm	-310	-421 to -199	<0.001

Supplementary Table 5: Multivariable model for age at device closure of patent ductus arteriosus (Indication of SBE prophylaxis only)

	Odds ratio	*95% CI*	*p*
Race/ethnicity			
Non-Hispanic white	1	n/a	n/a
Hispanic	1.0	0.58-1.65	0.94
Non-Hispanic black	1.3	0.80-2.21	0.27
Asian	1.9	0.95-3.87	0.07
Other	0.5	0.06-3.52	0.47
PDA anatomy			
Type A	1	n/a	n/a
Type B	2.2	0.6 to 7.4	0.21
Type C	4.1	2.6 to 6.5	<0.001
Type D	1.5	0.6 to 4.0	0.38
Type E	1.5	0.8 to 2.9	0.21
Hemodynamic vulnerability			
High Qp:Qs	3.2	2.1 to 4.8	<0.001
Low systemic arterial saturation	1.72	1.1 to 2.7	0.02
Elevated pulmonary vascular resistance	2.2	1.1 to 4.7	0.03

Supplementary Table 6: Multivariable model for composite major adverse events with device closure of PDA

	Beta	*95% CI*	*p*
Race/ethnicity			
Non-Hispanic white	1	n/a	n/a
Hispanic	95	-22 to 213	0.11
Non-Hispanic black	28	-108 to 164	0.69
Asian	283	47 to 520	0.02
Other	67	-249 to 383	0.68
Hemodynamic vulnerability			
Low systemic arterial saturation	-345	-450 to -239	<0.001
Low mixed venous saturation	-505	-653 to -358	<0.001
Right ventricular dysfunction	-45	-258 to 168	0.68
Dysplastic pulmonary valve	-317	-408 to -225	<0.001

Supplementary Table 7: Multivariable model for age (in days) of balloon pulmonary valvuloplasty

	OR	*95% CI*	*p*
Race/ethnicity			
Non-Hispanic white	1	n/a	n/a
Hispanic	1.6	1.1-2.4	0.02
Non-Hispanic black	1.2	0.7-1.9	0.56
Asian	3.2	1.6-6.3	0.0008
Other	1.2	0.4 to 4.1	0.76
Hemodynamic vulnerability			
Low systemic arterial saturation	1.5	1.0 to 2.1	0.04
Low mixed venous saturation	1.1	0.7 to 1.8	0.77
Dysplastic pulmonary valve	1.2	0.8 to 1.7	0.38

Supplemenary Table 8: Multivariable model for balloon pulmonary valvuloplasty referral with heart failure and/or right ventricular dysfunction

	OR	*95% CI*	*p*
Race/ethnicity			
Non-Hispanic white	1	n/a	n/a
Hispanic	0.9	0.5-1.6	0.77
Non-Hispanic black	1.4	0.8-2.6	0.22
Asian	1.6	0.6-4.5	0.40
Other	0.6	0.1-4.8	0.66
Hemodynamic vulnerability			
Low systemic arterial saturation	1.2	0.7-2.0	0.46
Low mixed venous saturation	2.4	1.4-4.1	0.002
Dysplastic pulmonary valve	2.2	1.4-4.6	0.001

Supplementary Table 9: Multivariable model for catastrophic adverse events with balloon pulmonary valvuloplasty

	Beta	*95% CI*	*p*
Race/ethnicity			
Non-Hispanic white	1	n/a	n/a
Hispanic	254	-93 to 602	0.15
Non-Hispanic black	472	-433 to 1377	0.31
Asian	-762	-1480 to -44	0.04
Other	-127	-992 to 738	0.77
Hemodynamic vulnerability			
Low systemic arterial saturation	-1,037	-1,375 to -698	<0.001
Bicuspid aortic valve	492	165 to 818	0.003

Supplementary Table 10: Multivariable model for age (in days) of balloon aortic valvuloplasty procedure

	OR	*95% CI*	*p*
Race/ethnicity			
Non-Hispanic white	1	n/a	n/a
Hispanic	2.6	1.5 to 4.5	0.0005
Non-Hispanic black	1.3	0.28 to 6.1	0.73
Asian	1.6	0.5 to 4.9	0.41
Other	2.0	0.6 to 7.3	0.29
Hemodynamic vulnerability			
Low systemic arterial saturation	1.8	1.1 to 3.0	0.03
Dysplastic pulmonary valve	0.8	0.5 to 1.4	0.45

Supplementary Table 11: Multivariable model for referral with heart failure and/or left ventricular dysfunction

	OR	*95% CI*	*p*
Race/ethnicity			
Non-Hispanic white	1	n/a	n/a
Hispanic	0.9	0.4 to 1.9	0.69
Non-Hispanic black	3.1	0.9 to 11.5	0.08
Asian	3.2	1.1 to 9.0	0.03
Other	0.8	0.1 to 6.2	0.8
Hemodynamic vulnerability			
Low systemic arterial saturation	1.0	0.5 to 2.0	0.91
Bicuspid aortic valve	0.5	0.3 to 1.0	0.04
Indication of LV dysfunction	2.9	1.3 to 6.5	0.009

Supplementary Table 12: Multivariable model for catastrophic adverse events after balloon aortic valvuloplasty

	OR	*95% CI*	*p*
Race/ethnicity			
Non-Hispanic white	1	n/a	n/a
Hispanic	1.1	0.6 to 2.1	0.77
Non-Hispanic black	3.2	1.0 to 10.0	0.05
Asian	2.3	0.8 to 6.2	0.11
Other	1/3	0.3 to 5.5	0.77
Hemodynamic vulnerability			
Low systemic arterial saturation	1.2	0.7-2.0	0.46
Bicuspid aortic valve	0.7	0.4 to 1.2	0.23
Indication of LV dysfunction	2.4	1.2 to 4.9	0.02

Supplementary Table 13: Multivariable model for composite adverse events after balloon aortic valvuloplasty

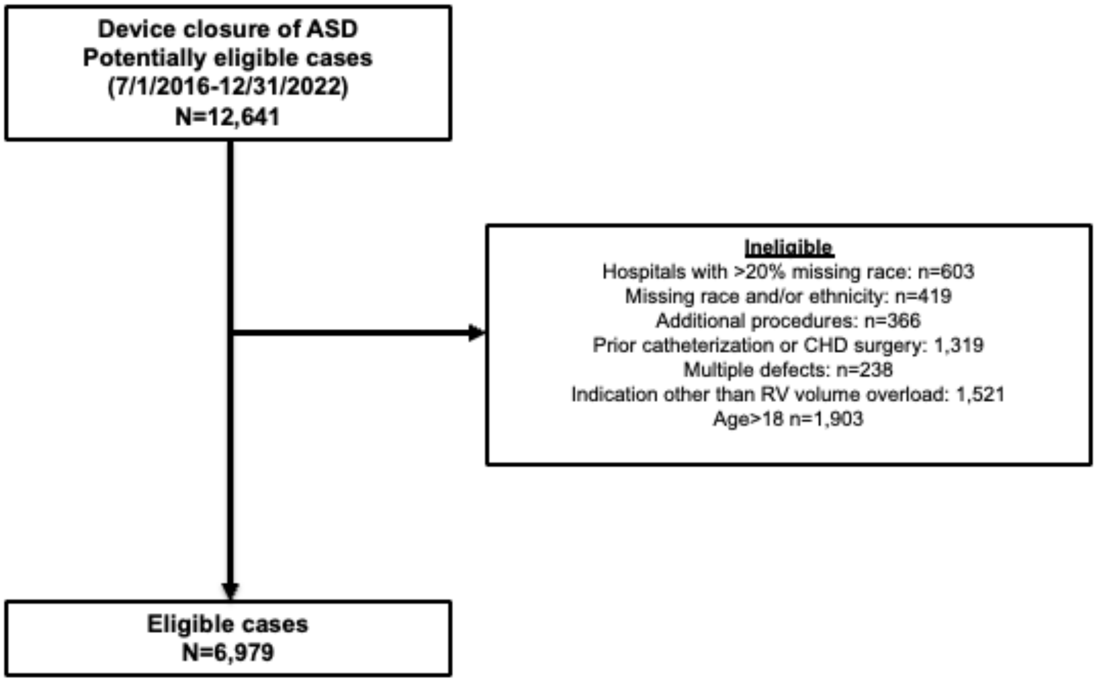

Supplementary Figure 1: Study population undergoing device closure of atrial septal defect

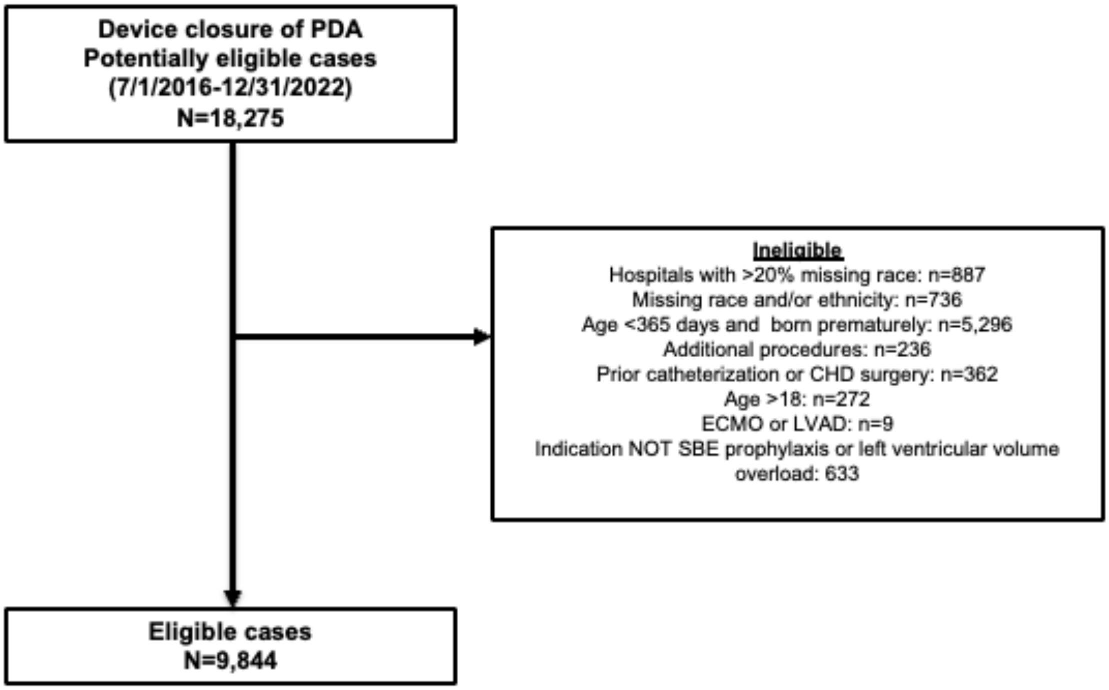

Supplementary Figure 2: Study population undergoing device closure of patent ductus arteriosus

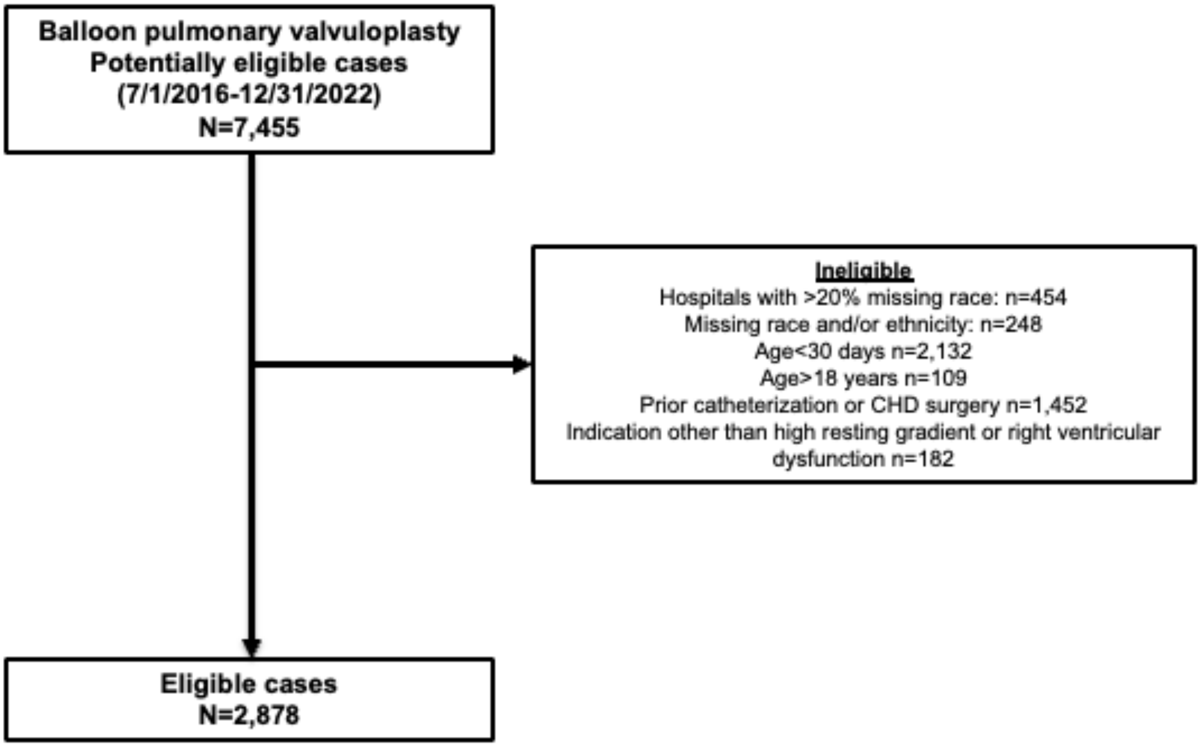

Supplementary Figure 3: Study population undergoing balloon pulmonary valvuloplasty

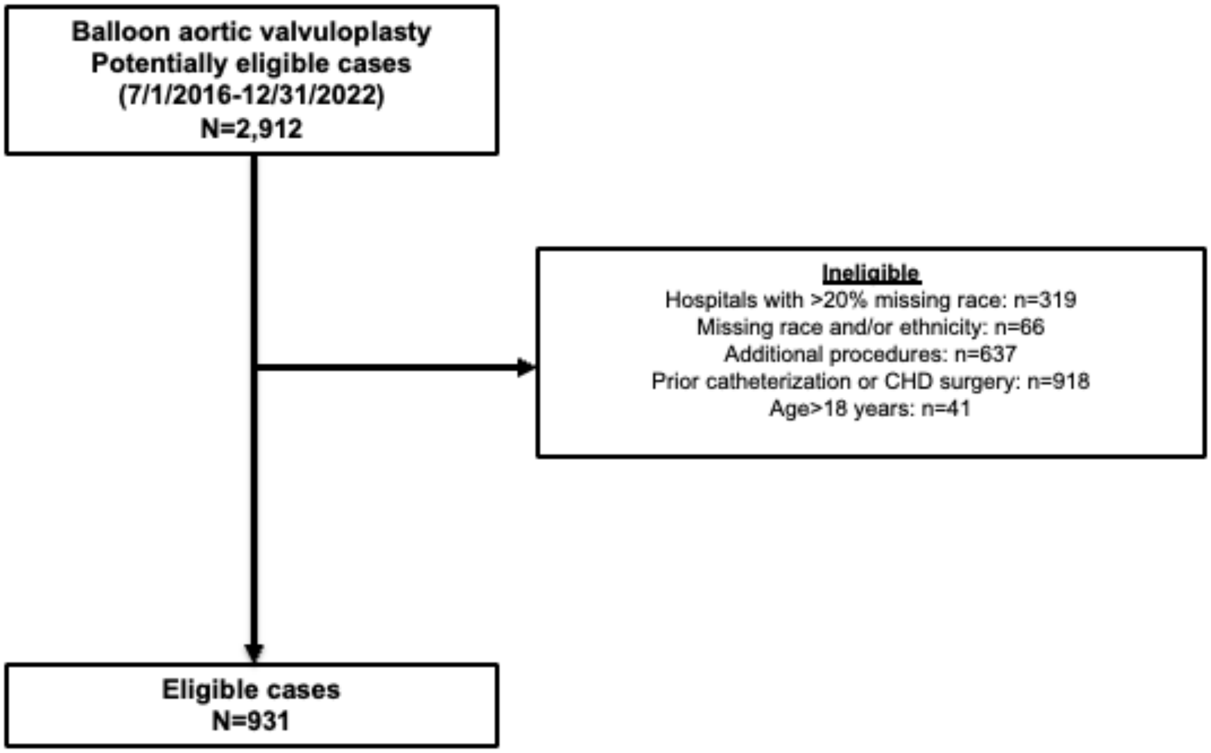

Supplementary Figure 4: Study population undergoing balloon aortic valvuloplasty
